# Daily Life Impacts and Desired Support for Mothers of Children with Sensory Integration Differences, with and Without Autism

**DOI:** 10.3390/brainsci16101105

**Published:** 2026-10-14

**Authors:** Susan Allen, Fiona Knott, Amanda Branson, Shelly J. Lane

**Affiliations:** 1 Department of Occupational Therapy, School of Sport, Nutrition and Allied Health Professions, Faculty of Health and Life Sciences, Oxford Brookes University, Oxford OX3 OFL, UK; 2 School of Psychology and Clinical Language Sciences, University of Reading, Reading RG6 6ES, UK; f.j.knott@reading.ac.uk (F.K.); a.branson@reading.ac.uk (A.B.); 3 Department of Occupational Therapy, College of health and Human Sciences, Colorado State University, Fort Collins, CO 80523, USA; shelly.lane@colostate.edu

**Keywords:** sensory integration, autism, family-centered practice, caregivers, occupation, everyday activities, relationships, media, parent intervention

## Abstract

**Highlights:**

**What are the main findings?**

- Mothers report that child sensory integration differences have an impact on many aspects of everyday life and relationships, with greater severity in some areas for autistic children.
- Mothers find positive relationships and media helpful and would like further support for themselves, their children, and greater education of others about sensory integration differences.

**What are the implications of the main findings?**

- Sensory integration differences and their impact on function and relationships should be considered whether a child has an autism diagnosis or not.
- Intervention strategies should consider media use and the development of supportive relationships.

**Abstract:**

**Background/Objectives**: To address the needs of mothers of children with sensory integration differences, we need a greater understanding of their experience and what they find helpful. This study aimed to explore the impact of child sensory integration differences on daily activities and relationships and to identify the relationships, media, and parent interventions mothers regard as helpful and further support that is desired. It compared perceptions of mothers of autistic and non-autistic children. **Methods**: A single cohort descriptive observational survey study. Questions were generated from previous in-depth interviews. **Results**: Mothers of autistic children versus non-autistic children both with sensory integration differences reported greater impact on school and community activities and on some relationships. No differences were observed in factors mothers identified as helpful, such as media resources and relationships. Most mothers in the full cohort reported that child sensory integration differences had a pervasive impact on everyday activities and relationships for both mother and child. Supportive relationships, media, social media, and direct interventions to mothers were helpful. Mothers wanted more practical and emotional support for themselves, education of others, and specialist support for their child. **Conclusions**: Mothers of children with sensory integration differences identified social and professional relationships, media, and parent intervention as helpful, supporting a variety of intervention strategies.

## 1. Introduction

Sensory integration differences [1] are uniquely identified as a component of the diagnostic criteria for autism [2]. This includes sensory hyper- or hypo-reactivity or unusual interest in the sensory aspects of the environment that interferes with social or occupational functioning. There is a diversity of terminology used related to the body of evidence generated from the initial work of A. Jean Ayres [3]. Acknowledging the variety of terms used, including sensory processing [4], sensory integration and processing, and the reported terminology used by autistic people [5], we have chosen to use the term sensory integration differences throughout this paper as originally described by Ayres [3].

Despite a lack of independent diagnostic recognition, there is increasing evidence that sensory integration differences occur both in autism and other neurodevelopmental populations, e.g., attention deficit hyperactivity disorder [6], developmental coordination disorder [7], and in individuals with no diagnosis [8]. Sensory integration differences vary across these neurodivergent profiles compared to neurotypical controls. Autistic children experience high rates of sensory modulation difficulties, prominently under-responsivity, followed by over-responsivity and sensory seeking [9,10], resulting in more prevalent challenges than those observed in children with DCD. Similarly, individuals with ADHD display significantly higher levels of atypical sensory integration difficulties across sensory sensitivity, sensory seeking, low registration, and sensory avoiding domains [11]. While children with DCD face greater modulation difficulties than their neurotypical peers, their sensory integration differences distinctly correlate with motor skills, a relationship absent in autistic children. Across both DCD and autism, heightened anxiety consistently correlates with sensory over-responsiveness [12]. Direct comparison of sensory integration difficulties across diagnostic groups and impact on daily occupations is limited [1]. Our interest is in autistic children because sensory integrative differences are diagnostic in this group. This contrasts with other neurodiverse groups that may experience sensory integrative challenges, but these challenges are not diagnostic. In the United Kingdom, pathways of care in the National Health Service are often based on diagnostic criteria in line with the National Institute for Health and Care Excellence guidelines [2]. This means that without an autism diagnosis, it can be difficult to access occupational therapy services. Therefore, it is important to contrast the impact of sensory integration differences on the occupations of everyday life between autistic and non-autistic children.

Occupations are made up of the activities that we participate in, and the link between a child’s sensory integration differences and successful participation in everyday activities for children and their families has been observed [13,14]. While occupational challenges are acknowledged [15], debate remains about how these issues are addressed, for example, through environmental adaptation or intervention with the child or caregivers [16,17]. The American Occupational Therapy Association advocates considering occupation across all environments [18], including physical, social, and attitudinal surroundings. Two research teams have explored approaches to working with autistic children with sensory integration differences and their families [16,17] in consideration of the environments in which their occupations take place. Ashburner et al. [16] described a clinical reasoning framework to optimize participation, dividing the model into support for the individual child or family, and support for multiple children in a shared environment. Reynolds et al. [17] took a different approach, conceptualizing interventions to support the functional goals of children with sensory integration differences by focusing on environmental support and adaptations, caregiver-focused interventions, and child-focused interventions. Both approaches recognize the need to support families, not just the child. Yet these frameworks are restricted to autistic children and their families. To support families whose children experience sensory integration differences that impact daily occupations with or without an autism diagnosis, we must have a greater understanding of their experiences.

Daily routines are shaped by culture and context [19]. Context includes both the physical structures of the environment and social relationships. The central context is the home environment, with the family or primary caregivers providing direct support. The next level of influence is created within the neighborhood and may include friends, extended family, schools, and leisure opportunities. Beyond this, political, economic, and social systems create influence on the child. Embedded within each of these levels are the societal beliefs and attitudes that guide behavior.

The family provides the most direct contextual influence for the child’s development. Mothers of children with sensory integration differences spend more time and energy in their parenting role [20] and adapt family life to make it more manageable, e.g., carefully selecting activities and adapting their environment [21]. The well-being of the mother has a direct impact on the well-being of the child [22]. However, the long-term stress experienced by mothers of children with sensory integration differences has been shown to be in addition to that associated with primary neurodevelopmental diagnosis [23,24]. Parental stress is known to be reduced by increased social support [25]. However, social support around parents of children with sensory integration differences has not been extensively studied to date, even though the link between family social support and parent stress has been established in wider populations [26]. Understanding the social networks around the family will help clarify how mothers of children with sensory integration differences perceive different forms of relationship support.

A systematic review of parent education and coaching for children with sensory integration differences found that intervention reduced parent stress and improved child occupations. Findings were limited by a small number of studies with differing methods and outcome measures, and a focus only on autistic children [27]. However, to our knowledge, no current literature identifies what mothers perceive to be helpful or the additional support they may want. In line with family-focused intervention, occupational therapists need to think creatively about how we can best support mothers, including understanding how mothers perceive the impact of intervention [16]. To start exploring development of preferred services, we previously interviewed six mothers of children with sensory integration differences [13]. Using qualitative thematic analysis, we grouped mothers’ experiences into two overarching themes. The first was ‘Impact on everyday life’ of the child’s sensory integration differences and subsequent challenges created at home, at school and in the community. The second theme identified ‘What helps;’ mothers felt empowered by gaining knowledge/understanding through others, media, supportive relationships, and being able to adapt activities and the environment. The ‘What helps’ theme supported participation in everyday life for both mothers and children, aligning with a multi-faceted intervention approach that includes direct support to parents, as described by Reynolds et al. [17].

The current study builds on the previous thematic study [13] by investigating the impact of maternal-reported child sensory integration differences on mothers’ and children’s everyday activities and relationships using a large community sample. Our secondary aim is to identify the relationships, media support, and parent interventions that mothers found helpful and to identify further areas in which they would value support. Comparing autistic individuals with non-autistic individuals will allow us to explore the wider experience of sensory integration differences in everyday life, therefore, data were analyzed by the subgroups of mothers of autistic children versus mothers of non-autistic children. Research questions were as follows:

1. a. What is the impact of sensory integration differences on child and mother everyday activities and relationships?
  b. Are there differences reported by mothers of autistic children and mothers of non-autistic children?
2. a. How do mothers rate the helpfulness of relationships, media support or parent intervention identified?
  b. Are there differences reported by mothers of autistic children and mothers of non-autistic children?
3. What additional support would mothers of children with sensory integration differences like to receive?

## 2. Materials and Methods

We initially examined this uncharted area using an exploratory and pragmatic approach, recruiting a single cohort of participants [28]. The ‘Strengthening Reporting of Observational Studies in Epidemiology’ [29] statement guided the development of this manuscript. Quantitative methods were used to identify current experience, and qualitative strategies were used to gain additional information. Data were gathered through a secure online survey tool to support ease of access across a wide population. Recruitment materials were shared through two online special interest sensory integration groups and one pediatric occupational therapy group, with an embedded link to the information sheet, consent form, and then survey. Ethics approval was granted by the Institutional Research Ethics Committee. Participants could withdraw from the survey until final submission, when the anonymized data were tabulated for analysis. We ran the survey between January and March 2020. Inclusion criteria required participants to be mothers of children with sensory integration differences who were residents of the United Kingdom or Ireland.

### 2.1. Survey Development

Our online survey was generated by the research team based on previous in-depth parent interviews [13]; all four authors collaborated in survey development. The survey was designed to gain further information in the areas identified by mothers in in-depth interviews; as such, formal psychometric evaluation was not undertaken. We piloted the survey with three mothers of children with sensory integration differences and two expert clinicians, who were asked to comment on clarity of questions and layout, leading to modifications to the wording and layout, e.g., tabulation of impact questions and splitting of choosing/participating in after-school or leisure activities. Sample size calculations were based on a 95% confidence interval and 5% margin of error, requiring a sample of 96 or more. Demographic data were collected on parent age, ethnicity, and level of formal education. Mothers were invited to provide data on all of their children with sensory integration differences. Child demographic data covered age, child’s formal diagnosis, and sensory integration classification as reported by mothers (Table 1). Sensory integration classification was based on the work of Bundy and Lane [1]. Likert scale questions were used to capture the perception of participants [30] about the impact of the child’s sensory integration differences on everyday activities and relationships, the helpfulness of relationships and resources and, where relevant, the content of parent intervention. Recognizing that not all children and mothers participate in all activities, we included a non-applicable option. Please see tables below for scaling. We used open-ended questions to identify additional resources or support mothers would like.

### 2.2. Data Analysis

Mothers’ responses were analyzed using IBM SPSS Statistics (Version 26; IBM Corp,. Armonk, NY, USA). The mothers were identified as mothers of an autistic child if one or more of their children presented with autism. For research questions 1 and 2, we described the full cohort by numbers (and percentage of respondents) reporting a negative impact and compared the group of mothers of autistic children to mothers of non-autistic children. The non-autistic group was composed of all mothers of children with no autism diagnosis; it included children with anxiety (23%), ADHD (17%), DCD (13%), as well as those with no identified diagnosis (28%). Descriptive statistics were calculated for quantitative data. Likert scales were transformed to numeric values. To explore impact in Table 2 and Table 3, no impact = 1, Minor negative impact = 2, Moderate negative impact = 3, Major negative impact = 4. To explore helpfulness in Table 4: 1. Unhelpful, 2. Not helpful, 3. Slightly helpful, 4. Very helpful, 5. Extremely helpful. This information is included in the footnote for Table 2, Table 3 and Table 4.

We used the Linear-by-Linear test (Chi-square test) to test for a trend in responses. To accomplish this, Likert scores were treated as categorical data. This analysis approach is known as the Mantel–Haenszel test or Cochran–Mantel–Haenszel statistics [31,32]. Two assumptions must be satisfied. The first is that the observations must be independent of one another, and the second is that the data must be acquired in the same manner for each group [32]. It is commonly used to understand relationships in observational studies while adjusting for potential confounders [33]. Significance was set cautiously at *p* < 0.01 *. We calculated effect size using Cramer’s V and interpreted it as follows: <0.2 reflects a weak effect, 0.2 to <0.6 is moderate, and >0.6 is strong. To control for the inflation of Type 1 errors due to multiple simultaneous statistical tests, the Benjamin–Hochberg (BH procedure was applied to the dataset of *p*-values. The false discovery rate was strictly set at Q = 0.01 (1%), bounding the expected proportion of falsely rejected null hypotheses among all significant discoveries to 1%. All *p*-values were sorted in ascending order and assigned individual ranks. Critical values = i/m × Q. Where i is the individual rank, m = total number of hypotheses tested, and Q is the false discovery rate. Out of the 44 hypotheses that were evaluated, the largest rank that satisfied the condition was determined to be *p* < 0.01. Indicating that at values of *p* < 0.01, the null hypothesis is rejected. Examining the data somewhat differently, Likert scale scores were treated as continuous data using descriptive methods (means and confidence intervals) [28]. Question 3 generated qualitative data. The qualitative data were categorized by two researchers and confirmed by a third, using basic content analysis [34].

## 3. Results

Completed surveys were analyzed (*n* = 132). A number of participants initiated the survey but did not complete it (*n* = 36), giving a completion rate of 79%. It was not possible to estimate the number of people who viewed the recruitment invitation. Please see demographic data in Table 1.

### 3.1. Everyday Activities and Relationships

#### 3.1.1. Research Question 1a: What Is the Impact of Sensory Integration Differences on Child and Mother Everyday Activities and Relationships?

For over two-thirds of all children (69%), mothers reported that sensory integration differences negatively impacted child and parent/family everyday activities at home, at school, and in the community (See Table 2). Almost all participants reported that mealtimes, morning/evening routines, child self-care, and choosing/participating in leisure activities were negatively impacted.

At least 68% of respondent mothers of children with sensory integration differences reported a negative impact on their own and their children’s relationships (See Table 3). For mothers, the impact was greatest with their partner. For children, the impact was greatest with peers.

**Table 2:** Reported impact of child sensory integration differences on mother and child everyday activities: Mothers of autistic versus non-autistic children.

Everyday Activities		Total Respondents Reporting a Negative Impact *n* (% of Respondents) ^a^	Mother of Autistic Children Mean (95% CI)	Mother of Non-Autistic Children Mean (95% CI)	*p*-Value ^b^	Linear by Linear Chi Squared (*df* = 1)	Effect Size Cramer’s V
At home	Family mealtimes	121 (92)	3.36 (3.14–3.59)	2.91 (2.66–3.16)	<0.01 *	6.94	0.28
	Family AM or PM routines	127 (97)	3.26 (3.07–3.45)	3.12 (2.91–3.34)	0.34	0.91	0.21
	Child self-care routine	121 (92)	3.32 (3.07–3.58)	3.06 (2.81–3.31)	0.14	2.17	0.21
	Child toileting	97 (74)	2.68 (2.40–2.96)	2.32 (2.03–2.65)	0.09	2.93	0.26
	Child being able to play independently	94 (71)	2.52 (2.13–2.74)	2.21 (1.76–2.28)	0.12	2.38	0.24
	Child Time spent on screentime	99 (78)	2.83 (2.52–3.07)	2.36 (1.99–2.58)	0.02	5.62	0.22
	Child—allocation of chores	99 (82)	2.97 (2.64–3.23)	2.57 (2.14–2.79)	0.07	3.33	0.24
	Childs sleep	112 (85)	3.21 (2.93–3.47)	2.91 (2.65–3.23)	0.12	2.37	0.17
	Parent sleep	102 (77)	3.03 (2.73–3.34)	2.48 (1.97–2.63)	0.01	6.62	0.29
	Family finances	106 (82)	3.22 (2.86–3.45)	2.50 (2.11–2.71)	<0.01 *	12.24	0.36
At school	Wearing school uniform	91 (78)	3.07 (2.76–3.31)	2.17 (1.92–2.47)	<0.01 *	19.38	0.41
	Dressing or undressing at school	94 (80)	3.07 (2.71–3.33)	2.14 (1.90–2.38)	<0.01 *	19.57	0.44
	Toileting at school	85 (69)	2.60 (2.14–2.80)	2.23 (1.82–2.42)	0.09	2.85	0.25
	Mealtimes at school	104 (83)	3.33 (3.01–3.63)	2.54 (2.15–2.76)	<0.01 *	13.82	0.35
	Time spent by parents dealing with school matters ^a^	114 (93)	3.41 (3.11–3.61)	2.73 (2.52–2.02)	<0.01 *	15.58	0.37
In the community	Choosing after school or leisure activities	113 (81)	3.65 (3.37–3.94)	2.98 (2.63–3.23)	<0.01 *	10.28	0.30
	Participating in after school or leisure activities	111 (90)	3.28 (3.02–3.54)	2.85 (2.56–3.14)	<0.01 *	9.35	0.30
	Toileting in the community	94 (72)	2.83 (2.45–3.10)	2.34 (1.93–2.56)	0.03	4.7	0.28
	Mealtimes in the community	121 (92)	3.42 (3.19–3.71)	2.93 (2.58–3.14)	<0.01 *	7.51	0.26
	Choosing family leisure activities	125 (95)	3.57 (3.36–3.82)	3.08 (2.80–3.30)	<0.01 *	8.61	0.32
	Being safe in community activities (e.g., not running away, not harming self or others)	108 (83)	3.29 (2.95–3.56)	2.55 (2.23–2.83)	<0.01 *	12.34	0.32

Legend: 1. No impact, 2. Minor negative impact, 3. Moderate negative impact, 4. Major negative impact. a. Not all participants responded to all questions. b. Rounded to nearest 2 decimal points, * *p* < 0.01.

**Table 3:** Reported impact of child sensory integration differences on mother and child relationships.

Relationship		Respondent Reporting a Negative Response *n* (% of Respondents) ^a^	Mother of Autistic Children Mean Responses (CI)	Mother of Non-Autistic Children Mean Responses (CI)	*p*-Value ^b^	Chi Squared (*df* = 1)	Effect Size Cramer’s V
Mother	Mother with partner	107 (91)	2.97 (2.70–3.16)	2.54 (2.35–2.87)	0.01	6.08	0.30
	Mother with family and extended family	106 (89)	2.94 (2.66–3.19)	2.45 (2.23–2.73)	<0.01 *	8.49	0.32
	Mother with her friends	105 (80)	2.73 (2.50–3.04)	2.12 (1.83–2.32)	<0.01*	11.32	0.30
	Mother with school staff	95 (75)	2.41 (2.15–2.71)	2.17 (1.89–2.41)	0.20	1.67	0.16
	Mother with other parents	103 (81)	2.61 (2.41–2.91)	2.20 (1.89–2.37)	0.01	6.17	0.24
	Mother within her community	85 (68)	2.53 (2.27–2.81)	1.73 (1.42–1.81)	<0.01 *	19.72	0.42
Child	Child with mother	99 (75)	2.48 (2.13–2.69)	2.18 (1.96–2.52)	0.09	2.90	0.16
	Child with family or extended family	115 (87)	2.79 (2.53–3.04)	2.35 (2.11–2.56)	<0.01 *	7.29	0.25
	Child with school staff	103 (76)	2.54 (2.32–2.86)	2.19 (1.92–2.41)	0.04	4.17	0.25
	Child with peers	125 (96)	3.38 (3.24–3.65)	2.81 (2.49–2.99)	<0.01 *	13.76	0.33

Legend: 1. No impact, 2. Minor negative impact, 3. Moderate negative impact, 4. Major negative impact. a. Not all participants responded to all questions. b. Rounded to nearest two decimal points. * *p* < 0.01.

#### 3.1.2. Research Question 1b: Are There Differences in Everyday Activities and Relationships Reported by Mothers of Autistic Children and Mothers of Non-Autistic Children?

There were significant differences that varied across environments in the severity of impact reported by mothers of autistic versus non-autistic children (See Table 2). At home, mothers of autistic children reported higher impact on family mealtimes and family finances. There were no reported differences in the remaining areas: self-care routines, toileting, play, time spent on screen, chores at home, although parents’ sleep approached significance. At school, mothers of autistic children reported higher impact on wearing uniform, dressing/undressing, mealtimes, and time spent by parents dealing with school matters. Only toileting at school did not demonstrate a significant difference between the subsamples. In the community setting, mothers of autistic children reported higher impact on being safe in the community, mealtimes, and choosing and participating in after-school and family leisure activities. Only toileting in the community did not differ significantly between the subsamples at the set level of significance. All everyday activities that reached significance showed a moderate effect size.

Despite the significant differences between subsamples across environments, both groups showed high rates of impact in all aspects of daily life addressed in the survey.

Greater negative impact of child sensory integration differences was reported by mothers of autistic children in mothers’ relationships with family, friends, and within their community. Impact on mothers’ relationships with their partner and other parents approached significance. The relationships that showed no significant difference at this level of significance between the two subsamples were the relationship between mother and child and between mother and school staff. With the caution of a weak effect size, this suggests that for mothers of children with sensory integration differences, relationships with school staff are negatively impacted in 75% of participants who responded, regardless of the presence or absence of an autism diagnosis (Table 3). Mothers reported that for autistic children versus non-autistic children, the impact of sensory integration differences was greater in family and peer relationships.

### 3.2. Helpfulness of Relationships and Media Resources

#### 3.2.1. Research Question 2a: What Is the Helpfulness of Relationships, Media Support or Parent Intervention Identified?

**Figure 1:**
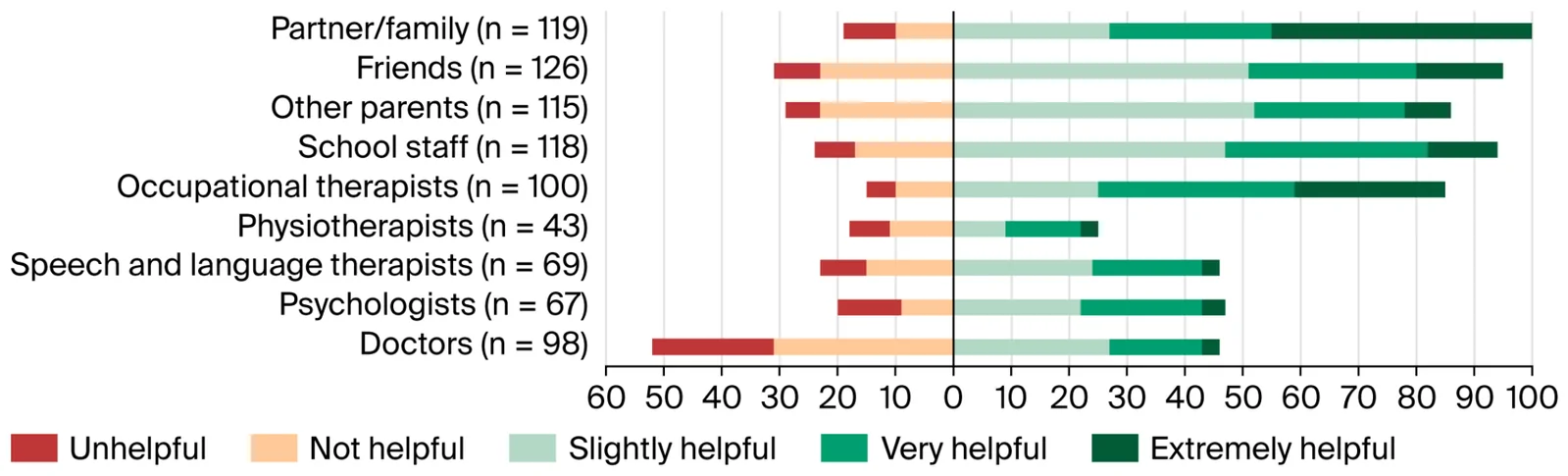
Helpfulness of relationships in providing practical or emotional support to mothers of children with sensory integration differences.

More mothers reported greater access to social/educational networks (friends, partner/family, other parents, and school staff) than health professional networks (occupational therapist, doctor, speech and language therapist, psychologist, and physiotherapists). Mothers were asked to identify the provision and helpfulness of practical or emotional support regarding their child’s sensory integration differences. See Figure 1. Mean ratings of helpfulness of relationships indicate that partners/family and occupational therapists were most often perceived to be ‘very helpful’; friends, other parents, school staff, physiotherapists, speech and language therapists and psychologists were generally ‘slightly helpful;’ and doctors were most often ‘not helpful’.

Mothers were asked if they accessed media resources (books, websites, social media) and to rate how helpful they found them. Websites (95%) and social media (94%) were most frequently accessed, followed by books (83%). Of those who accessed media resources more than 90% found them helpful. Please see Table 4.

**Table 4:** Comparison of helpfulness of relationships and resources comparing the experience of mothers of at least one autistic child with mothers of no autistic children.

Helpful Relationships and Resources (Number of Mothers of Autistic Children/Number of Mothers with Non-Autistic Children) ^a^	Respondent Reporting a Positive Response *n* (% of Respondents) ^a^	Mother of Autistic Children Mean (SD)	Mother of Non-Autistic Children Mean (SD)	*p*-Value ^a^	Chi Squared (*df =* 1)	Effect Size Cramer’s V
Relationships						
Partner and family (57/62)	100 (84)	3.67 (3.33–4.00)	3.84 (3.52–4.16)	0.46	0.56	0.12
Mum’s friends (63/63)	95 (75)	3.14 (2.86–3.42)	3.17 (2.92–3.43)	0.87	0.03	0.10
Other parents (57/58)	86 (75)	3.11 (2.84–3.37)	3.02 (2.78–3.26)	0.62	0.24	0.15
Occupational Therapist (54/46)	85 (85)	3.56 (3.24–3.87)	3.78 (3.46–4.11)	0.31	1.02	0.19
Physiotherapist (26/17)	25 (58)	2.96 (2.44–3.48)	2.71 (2.11–3.30)	0.50	0.45	0.29
Speech and Language Therapist (43/26)	46 (66)	3.02 (2.70–3.34)	2.73 (2.28–3.18)	0.27	1.22	0.26
Psychologist (43/24)	47 (70)	3.00 (2.64–3.36)	2.92 (2.42–3.41)	0.78	0.08	0.20
School staff (59/59)	94 (80)	3.39 (3.12–3.36)	3.08 (2.83–3.34)	0.10	2.65	0.20
Doctor (53/45)	46 (47)	2.53 (2.21–2.85)	2.42 (2.11–2.73)	0.63	0.23	0.25
Resources/Media						
Books (56/53)	103 (94)	4.09 (3.82–4.36)	3.89 (3.64–4.13)	0.27	1.24	0.20
Websites (63/62)	120 (96)	4.21 (3.98–4.43)	4.23 (4.03–4.42)	0.90	0.02	0.11
Social media (63/61)	122 (98)	4.19 (3.98–4.40)	4.20 (3.98–4.41)	0.27	1.24	0.20
Parent support groups (53/32)	80 (94)	4.00 (3.72–4.28)	4.28 (3.91–4.65)	0.22	1.54	0.20

Legend: 1. Unhelpful, 2. Not helpful, 3. Slightly helpful, 4. Very helpful, 5. Extremely helpful. a—Missing data excluded. * *p* < 0.01.

#### 3.2.2. Research Question 2b: Are There Differences Reported by Mothers of Autistic Children and Mothers of Non-Autistic Children?

When the sample was divided by mothers of autistic versus non-autistic children no significant difference in the helpfulness of relationships or media resources were reported. See Table 3.

#### 3.2.3. Usefulness of Parent Intervention

A subset of the total sample of mothers (*n* = 50) reported participation in intervention for parents of children with sensory integration differences. Within the sample of mothers receiving parent intervention, 66% had an autistic child and 34% were mothers of non-autistic children. Due to smaller sample sizes, no other comparison was made between mothers of autistic children and mothers of non-autistic children regarding parent intervention. This study did not explore the format of intervention, e.g., whether it was dyadic teaching or coaching.

Mean reported hours of intervention were 4 (Range 1–12). Most interventions were with groups of parents (73%) rather than individuals or family units (27%). Most were delivered by occupational therapists (84%), followed by speech and language therapists (18%) and physiotherapists (6%), teachers (4%), and other parents (4%).

19 (36%) of mothers reported goal-setting activities. Where mothers reported the goals, some were focused on performance (e.g., to stop his head banging) and others on participation (e.g., to be able to eat a meal together at home). Activities related to mealtimes were the most frequently reported goals.

Mothers rated the usefulness of topics covering types of sensory integration differences, the sensory systems, how sensory integration differences can affect everyday life, sharing experiences, practicing strategies, and practicing problem solving. On a 4-point Likert scale (not useful, a little useful, moderately useful, and very useful), all areas covered (except practicing problem solving) had a median and mode average of ‘moderately’ or ‘very useful’. Parent training covered most topics. Less frequently covered areas in parent training were the practical application of sensory strategies (72%) and problem solving (70%).

#### 3.2.4. Research Question 3: What Additional Support Would Mothers of Children with Sensory Integration Differences Like to Receive?

This section reports on the mothers (*n* = 64) who responded to this question. The data for autistic and non-autistic children were combined due to the smaller sample size. Mothers described the additional support they would like regarding their child’s sensory integration differences. Their responses were categorized into 3 themes (mothers’ words are in speech marks and participant number (P) is bracketed)

1. Practical and emotional support for mothers (*n* = 42): Mothers wanted information and support for their child’s participation in ‘daily routines’ (P28) across environments e.g., ‘how to’… ‘calm his body for sleep’ (P1) or ‘prevent sensory overload at football club’ (P44). Mothers wanted advocacy to navigate through services, e.g., Help ‘to talk to… staff’ (P32). They were looking for acknowledgment of their ‘resilience’ (P28) and to be ‘understood’ (P30).
2. Specialist support for their child (*n* = 17): Mothers advocated for access to specialist healthcare services and child leisure opportunities with an understanding of sensory integration differences, e.g., ‘occupational therapy’ (P42), ‘SLT’ (P108), ‘dieticians’ (P4), ‘dental services’ (P77), ‘sensory friendly leisure’ (P97) and ‘groups for children’ (P52)’. Mothers indicated that this access should be ‘regular’ (P53).
3. Education of others (*n* = 11). Training and support for school staff was identified as valuable ‘so that they can help him more’ (P6); as well as education of other professionals e.g., ‘doctors’ (P41) and ‘community staff’ (P68). The need to raise the ‘media profile’ as ‘the more people that have heard of it and have a general understanding the greater acceptance there will be for our children’ (P8).

## 4. Discussion

### 4.1. Impact on Everyday Life

In our full cohort, mothers reported a pervasive negative impact of their child’s sensory integration differences on everyday activities and relationships, for themselves and their children across home, school, and community environments. Consistent with the literature [21,35], child sensory integration differences impacted both children’s and mothers’ activities of daily living and leisure activities. Almost all mothers reported some level of impact on family mealtimes, morning and evening routines, and participation in activities in both school and community. When comparing mothers of autistic and non-autistic children, mothers of autistic children reported greater impact in the home, school, and community setting. At home, there is a significantly greater impact for autistic children at mealtimes and with family finances but not with morning/evening routines, child self-care, child toileting, child’s independent play, screen time, chores, child and parent sleep. At school, autistic children had a significantly greater impact on wearing school uniform, dressing/undressing, mealtimes, and time parents spend dealing with school matters, but not on toileting. In the community, significantly greater impact for autistic children was reported in choosing and participating in school or leisure activities, choosing family leisure activities and mealtimes in the community, but not toileting in the community. The data do not lend themselves to analyzing why these differences are more pronounced in the school and community setting. However, Law [36] states that participation in occupation is enhanced when the fit between the individual and the environment is optimized. We hypothesize that families of autistic children can provide greater adaptation in the home environment as opposed to the school or community environment.

The impact of child sensory integration differences on mothers’ health-promoting occupations was recently explored in a study of mothers of children aged 3–8 with and without sensory integration differences and no additional diagnosis [37]. Mothers of children with sensory integration differences reported reduced participation in health-promoting occupations and reduced quality of life. Our data, with these other findings, suggest a pattern of occupational imbalance in the reported experience of mothers of children with sensory integration differences, regardless of child autism diagnosis or not.

### 4.2. Impact on Relationships

Between 68% and 91% of respondents in the whole group reported that their child’s sensory integration differences negatively impacted their relationships with others, particularly with their partner and family. Mothers of autistic children reported a greater impact on their relationships with family, friends, and the community. However, child sensory integration differences appeared to impact relationships in many cases regardless of the child’s diagnosis. Acknowledging the heterogeneous nature of the non-autism group, it remains unclear whether autism features or sensory features drive these relationship challenges (See Section 4.6 for further comment).

The extent of the child’s sensory needs creates additional demands on the parents’ role; it therefore makes sense that the impact is felt within social relationships. Daly and colleagues [38] explored these demands in their qualitative evidence synthesis of family life with autistic children experiencing sensory integration differences. They reported that successful occupational engagement for the child depends on the ‘parent’s intense vigilance and forensic sense making to understand the child’s sensory life’ (p. 16). Impact on relationships may be exacerbated by the demands of the child or the lack of understanding within social relationships. Blaming mothers for child behavior in response to sensory integration differences is an internationally observed phenomenon [13,39], creating a negative cycle that reduces available support.

Social support serves as a buffer between parental stress and depression in parents of children with disabilities [25]. For parents of autistic children, the quality of interactive processes with partners and grandparents (specifically communication, listening, co-operation, support, and problem solving) determines their quality of life [40]. This aligns with our findings, where mothers report that supportive family relationships make a positive difference to everyday life. We believe strategies to enhance familial relationships warrant further exploration.

Child sensory integration differences most significantly affect the child’s relationships with family/extended family and peers. Cosbey and colleagues similarly reported reduced engagement with friends by children with sensory integration differences versus their matched typically developing peers [41]. Parents identify important goals for their children with sensory integration differences related to peer interaction for non-autistic children [42] and social participation for autistic children [43]. While we observe impact regardless of diagnosis, we do not yet fully understand how it affects the child. It may be that coping with sensory integration differences consumes the child’s resources, leaving less for developing peer relationships. Or that the observed behaviors deter peer relationships. We recommend further research in this area.

### 4.3. Relationship Helpfulness

Mothers of children with sensory integration differences experience higher parenting stress [24], making social support a critical determinant of health [44], so it is important to explore strategies to reduce that stress, e.g., strengthening family [26] and direct parent support. Our study showed that every relationship we asked about can help mothers, with partners and family offering the greatest reported helpfulness. This finding supports the value of family-centered practice. While this family-based approach has been explored with families of children with sensory integration differences [45], our study is the first to examine the relationships around mothers they consider supportive.

All health professionals except doctors were reported to be more helpful than not, a phenomenon Walbam [20] also observed. These findings should be applied with caution, as there was no discrimination in the data collected by the type of doctor, e.g., general practitioner (Family doctor) or pediatrician. Doctors may need more tools or information to help them understand the challenges of parenting a child with sensory integration differences, for example, ‘A decision guide for primary care providers’ [46]. Alternatively, there may be a mismatch in the mothers’ expectations of doctors as a resource [47]. Nonetheless, ways in which healthcare staff could be more helpful to mothers warrant further exploration.

Overall, mothers reported more access to social/educational support than to support from healthcare professionals and generally rated its effectiveness more highly. When we think about supporting the child, we often think about family-centered practice. However, our findings suggest there may be value in attending to a social network-centered care approach to fully capitalize on the family/friend support potentially available to mothers.

### 4.4. Media Resources

To our knowledge, this area has not been explored among mothers of children with sensory integration differences. As recruitment was online, the high rates of website and social media use are likely to have some level of bias. However, within this cohort, mothers report exceptionally high utilization and perceived helpfulness of websites and social media. This aligns with broader research with parents of children with physical disabilities, which notes that families often navigate a diverse ecosystem of information sources, actively comparing insights from healthcare professionals, peers, and websites at different stages of their care journey [48]. However, the academic literature regarding the utility of online health information remains highly polarized. On one hand, Daraz et al. [49] reviewed online medical information and noted wide variations in content quality, while Gibson [50] highlighted that parent of autistic children often viewed the internet as a less trustworthy source, favoring local access and peer group sharing instead. In contrast, Moon et al. [51] found that mothers of neurotypical infants highly valued the internet’s accessibility and anonymity, even reporting that online content could be more trusted than their immediate social or professional networks. We might hypothesize that parents will use internet resources up to a point but will seek more human-based connection as their demands increase.

Demographics also stratify digital resource engagement. A scoping review by Gruebner et al. [52] highlighted a tendency for younger, more tech-savvy parents to utilize these platforms more than individuals over 55 and those from lower socioeconomic backgrounds. Furthermore, Gruebner noted that research in this domain remains largely limited to more affluent nations. While the high accessibility of websites makes them an excellent vehicle for universal service delivery and basic information sharing [53], the lack of quality evaluation for health-related internet content presents an ongoing ethical challenge. As practitioners, we share a professional responsibility to guide clients toward accurate information and actively counter misinformation.

Mothers in our study reported frequent use of social media, highlighting a broader shift toward seeking experience-based knowledge. Research consistently shows that peers play a vital role in providing information and are often the preferred source for practical, lived-experience advice [48,52]. Social media elevates this dynamic beyond simple resource sharing by offering mothers a sense of community and perceived support in real time [54,55]. Consequently, healthcare providers must proactively harness social media to encourage healthy decisions [51]. For services supporting mothers of children with sensory integration differences, this reality presents both a distinct challenge and a powerful opportunity for future service delivery.

Books offer an alternative, more traditionally structured avenue for parents seeking to develop coping strategies, often benefiting from peer-review or editorial oversight. However, literature suggests that literature-based self-help cannot fully replace direct clinical intervention. When Schechtman et al. [56] directly contrasted professional coaching with self-help books for parents of children with ADHD, they found that while both methods yielded similar increases in parenting competence and improved parent-child interactions, the self-help book group had a significantly higher dropout rate. This may reflect the need for supportive relationships in therapeutic intervention.

Qualitative feedback from a mother in our study emphasized the critical need to raise the public media profile of sensory integration differences to foster greater societal understanding. Historically, occupational therapists have not been heavily engaged in macro- or society-level interventions. However, expanding our clinical perspective to include public advocacy and digital resource curation could yield substantial benefits for our clients. Ultimately, modern clinical approaches to supporting neurodivergent children and their families demand a greater, more adaptive variety of strategies that span far beyond the traditional clinic walls.

### 4.5. Parent Intervention

Mothers receiving direct intervention rated content highly, yet almost two-thirds of mothers in our study did not receive any intervention. We do not know if this was because services were not offered, or because mothers were not taking up services. In line with current evidence [27], mothers reported benefits of parent intervention, but the details of the specific characteristics that affected positive change remain unclear and require further investigation. The opportunity to work on problem-solving strategies using a coaching framework was less frequently included in available interventions. While parent education provides information, coaching enables a more active application of knowledge gained to everyday life [57]. Sharing information empowers choice, but active goal setting and problem-solving help individuals to shift from knowing to doing [58]. When goals are set in collaboration with the family, the focus of the intervention is meaningful to the client, clear, time/resources are used effectively, and outcomes are measurable. The practice of shared goal setting leads to better achievement of outcomes [59]. About 36% of mothers in our study reported goal setting as part of the parent intervention, suggesting this strategy should be used more to effect positive change for families.

When identifying further sources of support, mothers reported wanting ‘practical and emotional support’, ‘specialist support for their child’, and the ‘education of others to promote understanding’. From a childhood disability perspective, the ecological framework [60] helps to identify several modifiable factors that support the child’s participation through engagement with families, communities, and social systems [61]. At the center of the ecological framework sits ‘specialist support to the child’. The efficacy of direct child occupational therapy intervention is debated [62,63] in this population, but it is recognized that intervention leads to gains in individualized goals [64] and continues to be identified as a need by mothers in this study. This may reflect tension between what parents want and the services available to them in this study’s geographical context. The shift to the next ecological stage of family focus is supported by mothers wanting ‘practical and emotional support’. Evidence to date suggests that occupational therapists share information through parent education and are beginning to take a more proactive approach in supporting families to problem-solve themselves through individual family coaching [27,65]. However, while individual family coaching builds resources within the family, it does not take the next ecological model step into wider community engagement. Mothers in our study identified the ‘education of others’ as key, extending an understanding of the need to engage with their own community but also create an influence at societal level, e.g., ‘through use of media’. Parent intervention is one intervention in a range of strategies to help children with sensory integration differences and their families in everyday life.

### 4.6. Strengths, Limitations, and Future Directions

The study has several strengths, including its focus on mothers’ experiences in a geographical population not previously explored. No other studies have sought to quantify the extent of impact on everyday life for mothers and their children with sensory integration differences. No other studies have explored the impact on relationships. No other studies have asked mothers what they want. Future studies may wish to explore these issues with other caregivers, e.g., fathers. Ethnicity and mothers’ educational attainment were limited, potentially reducing generalizability, and future studies should consider them. Data were collected online. As noted, this may have biased the data, as respondents would be more likely to be those engaged in social media and potentially sensory-focused resources. In addition, data were collected prior to and into week one of the initial COVID-19 lockdown, involving 4 respondents. Descriptively, these cases matched the cohort and were therefore included in the data collection. The shift from face-to-face to online services may impact longer-term findings on parent intervention.

Participants were asked to consider the survey questions over the last 12 months. Each mother reported on up to 3 of her children. The mothers were identified as mothers of an autistic child if one or more of their children presented with autism. Future studies may wish to seek data on one parent and one child only; this would allow inspection of the link between types of sensory processing differences more specifically to challenges in daily life and impact on relationships. Mothers’ demographics were similar in both groups. Child demographics demonstrate some differences, with higher rates of sensory integration difficult. We recommend further exploration of the links between the classification of sensory integration difficulty and the impact on function across diagnosis.

We did not formally evaluate the psychometric values of the survey although survey questions were based on prior parent interviews. Additionally, parent and expert clinician feedback was gained and changes made as suggested. Use of a content validity index with content experts would have strengthened the study.

As an exploratory study, we established diagnosis through parent report rather than full screening. The non-autistic group combined children with a range of diagnoses (Anxiety, ADHD, DCD) and no diagnosis, all with mother-reported sensory integration differences. It is acknowledged that clinically distinct populations differ in terms of sensory presentation and family impact. We recommend future studies with a larger, fully assessed sample and a comparator group without sensory integration differences to more specifically test whether the identified patterns hold up. Data would be further strengthened by a power calculation tied to the group comparison effect sizes.

Because we were interested in comparing autistic children with sensory integrative differences to other children also having sensory integrative differences, we did not have a typical comparison group. As such, it is feasible that some of the challenges reported by mothers could be due to factors other than those related to sensory integration differences. However, differences that we identified have been reported by other investigators and linked to sensory integration differences [20,21,38,41]. Nonetheless, additional research is warranted.

Future studies can explore the impact on occupation and relationships in those with and without sensory integration differences as defined by available assessment tools (e.g., parent reports of child sensory integration differences, standardized testing). Future studies should also examine the links between child functional concerns, relationship impacts, and the types of support most helpful to mothers. Finally, these data focus only on mothers; we recognize that not all primary carers are mothers. Future research would benefit from expanding inclusion criteria to encompass the views and needs of all primary carers.

## 5. Implications for Practice and Conclusions

Mothers report a pervasive impact on daily activities and relationships of reported child sensory integration differences for both parent and child. The reported impact on everyday activities and relationships is greater in mothers of autistic children. Most mothers, regardless of diagnosis, find supportive social and professional networks and media helpful. Direct intervention is perceived as useful. Mothers advocate for further practical and emotional support for themselves, specialist support for their child, and education of others. We argue that occupational therapists should consider:

- Building social and professional networks with an understanding of the impact of sensory integration differences on daily life.
- Providing direct intervention to mothers, including goal setting and practicing problem-solving, e.g., coaching.
- Expanding the use of carefully curated online resources and social media to share information and support advocacy for children with sensory integration differences and their mothers.

## Figures and Tables

**Table 1:** Demographics.

			Autism Subgroup (*n* = 67)	Non-Autism Subgroup (*n* = 65)	Total Sample
Mothers (*n* = 132)	Mean age (years)		42	38	39
	Ethnicity (%)	White	99	96	99
		Mixed/multiple	1	1	2
		Asian/Asian British/Asian Irish		1	1
		Black/African/Caribbean/ Black British/Black Irish			-
		Other ethnic groups			-
		Prefer not to say		1	1
				
	Highest level of education (%)	Postgraduate	18	33	
		Degree or equivalent	34	28	27
		Higher education	18	16	33
		A level or equivalent *	7	12	18
		GCSE or equivalent **	13	7	11
		Other qualifications	7	2	11
		No qualifications	-	1	5
		Prefer not to say	6	1	1
					
Children (*n* = 175) ***			Autism (*n* = 77)	Non autism (*n* = 98)	
	Mean age (years)		10	8	9
				
	Gender (*n*)	Male	48	44	66
		Female	19	20	33
		Non-Binary		1	1
				
	Classification **** (%)	Sensory over-responsivity	61	34	61
		Sensory seeking	52	38	58
		Poor body awareness	38	23	39
		Sensory under-responsivity	31	23	32
		Poor tactile perception	27	18	30
		Sensory based dyspraxia (Somatosensory)	4	6	27
		Sensory based dyspraxia (Vestibular Bilateral and sequencing)	14	16	15
		Inadequate interoception	23	16	26
		Poor visual perception	23	12	23
		Poor discrimination	10	7	11
		Don’t know	6	6	6

N.B. Numbers are rounded to the nearest whole number. * Examinations at the end of optional additional schooling, generally taken at age 18 years. ** Examinations at the end of statutory schooling, generally taken at age 16 years. *** Parents reported on up to 3 children each. **** Responded to more than one category, Classification according to Bundy and Lane [1].

## Data Availability

The data presented in this study are available on request from the corresponding author, as they are part of a larger dataset.
